# Circulating microRNAs in patients with intracranial aneurysms

**DOI:** 10.1371/journal.pone.0176558

**Published:** 2017-05-01

**Authors:** John A. L. Meeuwsen, Femke N. G. van ´t Hof, Wouter van Rheenen, Gabriel J. E. Rinkel, Jan H. Veldink, Ynte M. Ruigrok

**Affiliations:** Department of Neurology and Neurosurgery, Brain Center Rudolf Magnus, University Medical Center Utrecht, The Netherlands; Stellenbosch University Faculty of Medicine and Health Sciences, SOUTH AFRICA

## Abstract

**Introduction:**

We compared circulating microRNA (miRNA) levels in plasma of patients with intracranial aneurysms (IA) to those of controls as a first step towards finding potential biomarkers for individuals at high risk of IA development and its subsequent rupture.

**Patients and methods:**

Using a PCR array we measured 370 miRNAs in plasma of 15 patients with prior aneurysmal subarachnoid hemorrhage (aSAH), of whom 11 had an additional unruptured IA (UIA), and of 15 controls. MiRNAs with a difference in levels with an absolute fold change (FC) > 1.2 and p<0.01 were further tested using real-time (RT) PCR in an additional independent set of 15 aSAH patients, 15 untreated UIA patients and 15 controls for replication (absolute FC >1.2 and p<0.05). We used receiver operating characteristic (ROC) curves to illustrate the diagnostic potential of these miRNAs.

**Results:**

Three of five miRNAs with a difference in levels in the PCR array study were replicated with miRNA-183-5p decreased in all patients (FC = -2.2, p = 1.7x10^-3^), miRNA-200a-3p increased in aSAH patients (FC = 1.8, p = 2.8x10^-2^) and miRNA-let7b-5p decreased in UIA patients (FC = -1.7, p = 1.27x10^-3^) as compared to controls. In distinguishing aSAH patients from controls, the area under the ROC curve (AUC) was 0.80 (95% confidence interval (95% CI) 0.63–0.97) for miRNA-183-5p, and 0.74 (95% CI 0.55–0.94) for miRNA-200a-3p. In distinguishing untreated UIA patients from controls, AUC was 0.83 (95% CI 0.69–0.98) for miRNA-183-5p and 0.92 (95% CI 0.81–1) for miRNA-let-7b.

**Discussion/Conclusions:**

We identified three specific circulating miRNAs that are able to discriminate between IA patients and controls. Follow-up studies should assess if these miRNAs may be used biomarkers for identifying individuals at high risk of IA development and its subsequent rupture.

## Introduction

Subarachnoid hemorrhage from a ruptured intracranial aneurysm (aSAH) is a severe subtype of stroke, occurring in relatively young people (mean age 50 years), of whom a third dies as a consequence of the aneurysmal SAH (aSAH).[1] Individuals with a positive family history for aSAH—i.e. having ≥2 affected first-degree relatives with aSAH—have a substantial risk of intracranial aneurysms (IA) and life-time risk of aSAH.[2,3] Screening with MR angiography and preventive treatment of identified IA in such persons decreases the risk of aSAH and is cost-effective.[4] Other groups have been identified with an increased life-time risk of aSAH, but not to the same extent as those with ≥2 affected first-degree relatives. These include patients with one affected first-degree relative, former patients who have survived an episode of aSAH, and persons with a high burden of environmental risk factors.[2,5,6] In these groups of patients, screening with MRA is less or not cost-effective.[7–9] The inefficacy of screening using MRA to identify all individuals at high risk stresses the need for new screening methods to improve detection of individuals with high risk of aneurysmal development or rupture. Biomarkers in blood may be such a method. MicroRNAs (miRNAs) circulating in blood have been proposed as potential biomarkers, especially since they are stable in plasma and serum.[10] MiRNAs are small, approximately 22 nucleotides long, single-stranded RNA molecules that play an important role in the post-transcriptional regulation of gene expression.[10] Altered circulating miRNAs levels have been reported for numerous cardiovascular disorders.[11,12]

Given the high, long-term risk of developing a new IA in patients with priors aSAH,3 IA seems to reflect a chronic disease of the vessel wall. We, therefore, hypothesized that altered miRNA profiles are present during life in IA patients, also in those in whom the IA has been occluded. We compared miRNA profiles in plasma of patients with a past aSAH to those in controls. MiRNAs with a difference in levels were replicated in an independent set of aSAH patients and controls. Furthermore, in this replication study, we assessed whether the miRNAs with a difference in levels are also able to distinguish untreated, unruptured IA (UIA) patients from controls. Our aim in investigating these miRNA profiles was to make a first step towards the development of potential biomarkers for individuals at high risk of IA development and its subsequent rupture.

## Methods

### Study design and patient samples

#### Discovery study using PCR array

For our current miRNA analysis, we randomly selected 15 patients and 15 controls participating in a genome-wide expression study (GWES) in blood of 119 aSAH patients and 118 healthy controls. Patients in this GWES were admitted to the Department of Neurology and Neurosurgery of the University Medical Center Utrecht (UMCU) between 1999 and 2007.[13] For the GWES, only patients who had had their aSAH at least two years prior to blood sample collection were included in order to minimize the chance of detecting direct effects of the hemorrhage on expression profiles. aSAH was defined by clinical symptoms indicative of aSAH combined with blood on a CT scan and a proven aneurysm at angiography (catheter, CT- or MR angiogram). Only patients with complete occlusion of the ruptured IA were included. Of the selected 15 aSAH patients, 11 had additional UIA, three of which were left untreated. Controls were mostly spouses of the aSAH patients included in the GWES. Controls confirmed a negative history of aSAH or UIA by interview.

#### Replication study using real-time (RT) PCR

For an independent replication cohort, we randomly selected an additional sample of 15 aSAH patients and 15 healthy controls to verify miRNAs with a difference in levels discovered in the PCR array study. In addition, we selected a sample of 15 patients with untreated, UIA to assess whether the miRNAs with a difference in levels established in the discovery study are also able to discriminate this type of patient from controls. The aSAH patients and controls were also randomly selected from the previous GWES, whereas the untreated UIA patients were selected from our prospectively collected database which includes blood samples of patients with intracranial vascular malformations. The approach used for blood sample collection and processing for both the aSAH and UIA patients was identical. Information was collected on age, gender, smoking history and presence of familial aneurysms (≥2 affected first-degree relatives with aSAH) for all patients and controls. We excluded patients with other cardiovascular diseases and/or cancer in their past medical history since these diseases may influence the levels of miRNAs in our patients. The medical ethical committee of the University Medical Center Utrecht approved the study. All participants provided written informed consent.

### Blood sample collection and processing

Plasma was obtained from blood in EDTA tubes by centrifugation at 1200 x g for 10 minutes at room temperature, and stored at -80°0°C until further use. MiRNAs were isolated from plasma using the miRNeasy Serum/Plasma Kit (Qiagen, Germany), according to the manufacturer’s protocol. Pre-amplification was performed using the miScript PreAMP PCR Kit (Qiagen) to obtain sufficient amounts of cDNA for miRNA analysis with real-time (RT) PCR.

#### Discovery study using PCR array

RT-PCR was performed using a MIHS-3106Z PCR array, which is a plasma- and serum-specific PCR array (Qiagen) that delivers a panel of 370 miRNAs detectable in serum and plasma. For all 30 samples of the patients and controls, we measured levels of the miRNAs and five housekeeping genes (miRNA-15b-5p, miRNA-126-3p, miRNA-21-5p, miRNA-30c-5p and miRNA-148b-3p) in triplicate, based on SYBR green fluorescence as detected by the ViiA^™^ 7 Real-Time PCR System.

#### Replication study using RT PCR

The significantly miRNAs with a difference in levels defined in the PCR array study (see paragraph ‘Statistical analysis’) were further analyzed with RT-PCR using single primer sets for the miRNAs and the five housekeeping genes. The 45 samples of patients and controls were analyzed in triplicate using SYBR green fluorescence-based detection.

#### Statistical analysis

**Fig 1:**
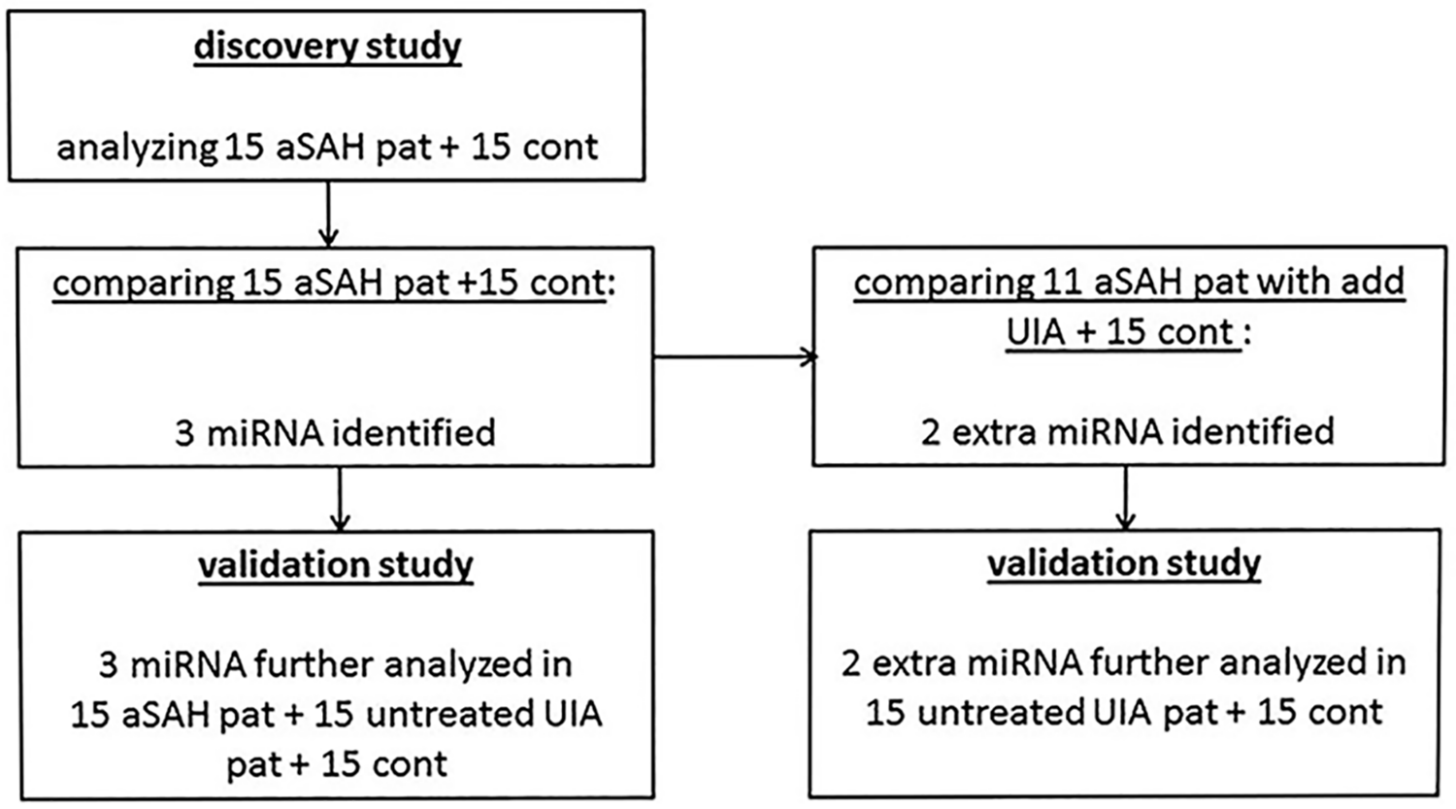
Flow diagram of miRNA analyses in discovery and replication study. aSAH, aneurysmal subarachnoid hemorrhage; pat, patients; UIA, unruptured intracranial aneurysm; cont, controls; add, additional.

miRNA with a difference in levels Statistical analyses were performed using version 3.0.1 of the R Statistical Software.[14] The cycle threshold (Ct-)values of all miRNAs in all patients and controls were normalized by subtraction of the average Ct-value of the five housekeeping miRNAs resulting in dCt values. Then differences in miRNA levels between patients and controls were assessed comparing these dCt values of patients to those of controls using the delta delta Ct method.[15] In the PCR array study, we first compared dCt values using this delta delta Ct method of all aSAH patients to controls using logistic regression with adjustment for sex. Secondly, we compared dCt values of the 11 aSAH patients who had additional UIA to controls. From these analyses, we selected the miRNAs yielding the most significant difference in levels as defined by an absolute fold change (FC; calculated as 2^-(dCt patient – dCt control)^) > 1.2 or < 0.8 and p < 0.01 in the PCR array study. These selected miRNAs were further analyzed in the replication study. In this replication study, we first compared all 30 patients with controls. In a second analysis, we compared the 15 aSAH patients to controls and separately the 15 UIA patients to controls. The miRNAs that were only found to have a difference in levels when comparing aSAH patients with an additional UIA to controls in the PCR array study, were further analyzed in the 15 untreated UIA patients and the 15 controls (and not in the 15 aSAH patients) in the replication study (Fig 1). We considered miRNAs with an absolute FC > 1.2 or < 0.8 and p < 0.05 to have significant difference in levels in the replication study.

#### Receiver operator characteristic curves

For the miRNAs with a significant difference in levels, receiver operator characteristic (ROC) curves were established applying the pROC package in R to explore the diagnostic potential of these miRNAs as biomarkers. The model was trained with z-values of the miRNAs in the PCR array study and tested with the z-values from the replication study. The predictive value of the ROC curve was expressed as the area under the curve (AUC) with corresponding 95% confidence intervals (95% CI) and the optimal Youden’s J thresholds. In addition, we reported the confusion matrices of the models. For the analysis on distinguishing aSAH patients from controls we analyzed both the single miRNAs identified and the combination of these miRNAs together. We repeated this same analysis for the distinction of patients with untreated UIA from controls.

#### Sample size calculation

The sample size for the PCR array study was calculated based on the following assumptions: 370 miRNAs under study, an estimated two-fold change in miRNA level difference with a standard deviation of 0.7 on the basis of an alpha of 5% and a power of 80%.[16] In the replication study, we calculated the sample size using the lowest effect size and standard deviation values of the miRNAs with a difference in levels as established in the PCR array study, an alpha of 5% and a power of 80% in a one-sided t-test.

### Target genes of miRNA

Next, we identified potential target genes of the miRNAs with a difference in levels. We selected a list of 109 candidate genes with a previous described association with IA in the literature consisting of all genes in significant loci from previous genome wide association studies (GWAS) (n = 52) [17,18] and genes differentially expressed in IA tissue as compared to control tissue in at least two GWES (n = 57).[19] Subsequently, we searched for overlap between this list of 109 candidate genes and the target genes of the miRNAs with a difference in levels. Target genes of these miRNAs were identified using MiRecords.[20] We selected miRNA target genes which were experimentally validated, or predicted by at least four prediction programs. We assessed the probability that the number of overlapping genes was equal or higher than would be expected by chance using hypergeometric testing assuming that a total of 20,500 genes are present in the genome [21] and the number of overlapping genes between the target genes of the miRNAs and the list of 109 candidate genes follows a hypergeometric distribution.

## Results

### Baseline characteristics

The baseline characteristics of the patients and controls of the miRNA PCR array and replication study are shown in Table 1.

**Table 1:** Baseline characteristics of patients and controls.

	Discovery study		Replication study		
Characteristics	aSAH cases	controls	aSAH cases	UIA cases	controls
Total number	15	15	15	15	15
Mean age (range)	56 (45–72)	53 (44–65)	57 (47–67)	56 (34–79)	58 (31–75)
Women *N* (%)	11 (73)	11 (73)	11 (73)	9 (60)	0 (0)
FIA *N* (%)	0 (0)	0 (0)	0 (0)	2 (13)	0 (0)
History of smoking *N* (%)	12 (80)	11 (73)	15 (100)	8 (53)	12 (80)
Cases with additional aneurysms *N* (%)	11 (73)	NA	1 (7)	5 (33)	NA

aSAH, aneurysmal subarachnoid hemorrhage; UIA, unruptured intracranial aneurysm; FIA, familial intracranial aneurysm, N, number; NA, not applicable.

### Discovery study

We found three miRNAs with a difference in levels between aSAH patients and controls: miRNA-200a-3p (FC = 1.6, p = 2.2x10^-4^), miRNA-183-5p (FC = -1.6, p = 3.9x10^-3^) and miRNA -2355-3p (FC = 1.3, p = 5.5x10^-3^) (Table 2). In the sub-analysis comparing only the 11 aSAH patients with additional UIA to controls, two additional miRNAs, miRNA -141-3p (FC = 1.7, p = 9.9 x10^-4^) and miRNA -let-7b-5p (FC = -1.4, p = 5.4 x10^-3^) were identified (Table 2).

**Table 2:** MiRNAs with a difference in levels between patients aSAH and controls in the PCR array study.

	aSAH cases (n = 15) vs. controls (n = 15)		aSAH cases with UIA (n = 11) vs. controls (n = 15)	
miRNA	fold change	p-value	fold change	p-value
200a-3p	1.6	2.2x10^-4^	1.8	8.9x10^-5^
183-5p	-1.6	3.9x10^-3^	-2.3	1.3x10^-3^
2355-3p	1.3	5.5x10^-3^	1.5	1.8x10^-3^
141-3p	1.4	1.9x10^-2^	1.7	9.9x10^-4^
Let7b-5p	-1.1	8.5x10^-2^	-1.4	5.4x10^-3^

aSAH, aneurysmal subarachnoid hemorrhage; UIA, unruptured intracranial aneurysm

### Replication study

In the replication study, miRNA-183-5p level was significantly lower in all 30 IA patients combined as compared to the controls (FC = -2.2, p = 1.7x10-3), and also in the subsets of 15 aSAH patients (FC = -2.1, p = 4.1x10-3) and 15 UIA patients (FC = -2.4, p = 1.3x10-2) as compared to the controls (Table 3). MiRNA-200a-3p level was significantly higher in aSAH patients than in controls (FC = 1.8, p = 2.8x10^-2^) (Table 3). MiRNA-let-7b-5p was significantly lower in untreated UIA patients as compared to controls (FC -1.7, p = 1.3x10^-3^). The increase of miRNA -2355-3p and miRNA-141-3p as identified in the PCR array study could not be confirmed.

**Table 3:** MiRNA-200a-3p, miRNA-183-5p, miRNA-2355-3p, miRNA-let7b-5p and miRNA-141-3p in patients with aSAH and / or UIA as compared to controls in the replication study.

	all cases (n = 30) vs. controls (n = 15)		aSAH cases (n = 15) vs. controls (n = 15)		UIA cases (n = 15) vs. controls (n = 15)	
miRNA	fold change	p-value	fold change	p-value	fold change	p-value
200a-3p	1.4	0.14	**1.8**	**2.8x10**^**-2**^	1.1	0.42
183-5p	**-2.2**	**1.7x10**^**-3**^	**-2.1**	**4.1x10**^**-3**^	**-2.4**	**1.3x10**^**-2**^
2355-3p	-1.2	0.16	-1.1	0.26	-1.2	0.16
141-3p	ND	ND	ND	ND	-3.5*	6.9x10^-3^
Let7b-5p	ND	ND	ND	ND	**-1.7**	**1.3x10**^**-3**^

*FC has an opposite direction as compared to the direction of FC found in PCR array study (FC = 1.7)

ND, not determined; aSAH, aneurysmal subarachnoid hemorrhage; UIA, unruptured intracranial aneurysm; FC, fold change

### ROC curves

**Fig 2:**
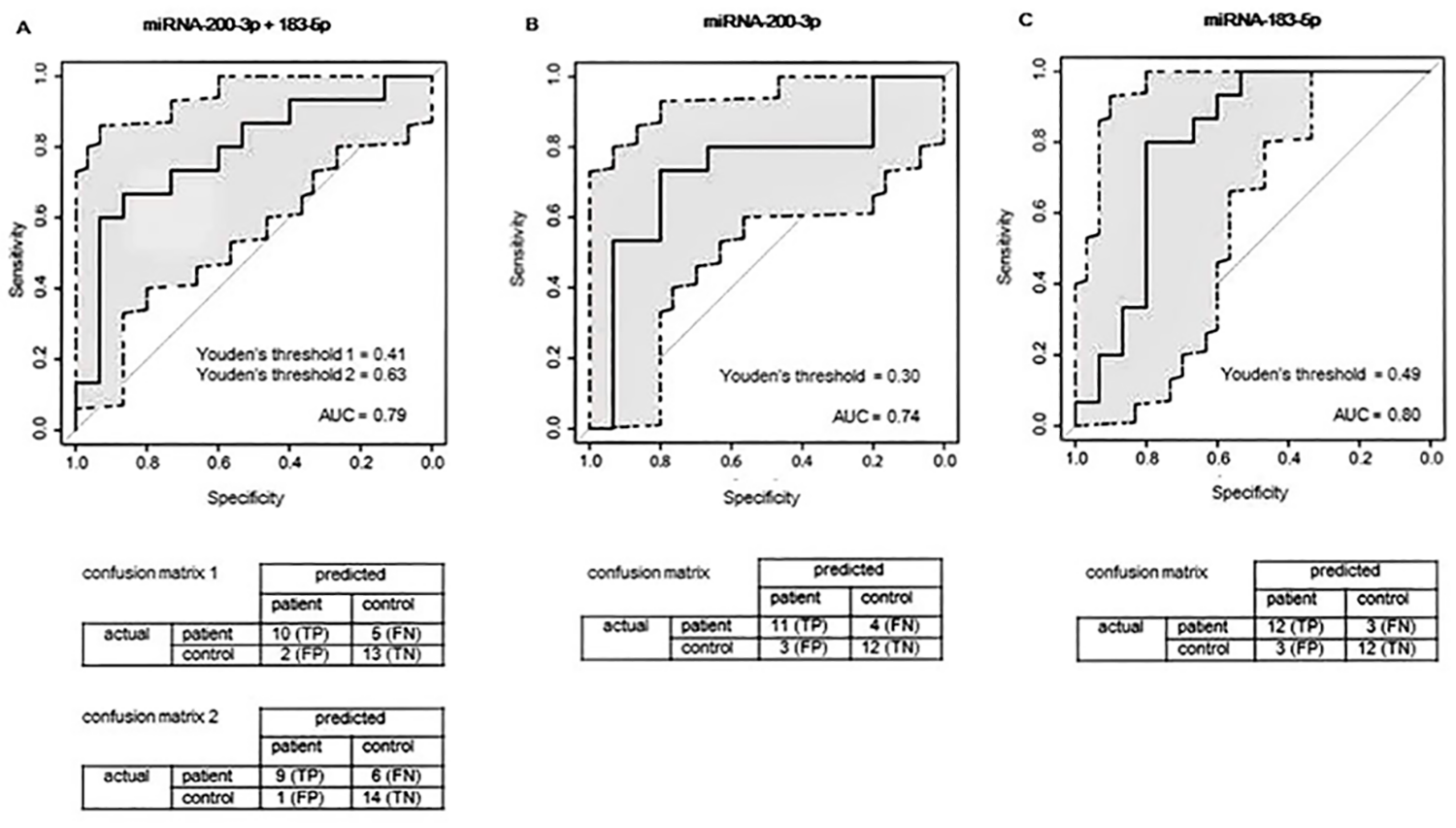
ROC curves of patients with aSAH and controls. ROC, Receiver operating characteristic; aSAH, aneurysmal subarachnoid hemorrhage; AUC, area under the curve; TP, true positives; FN, false negatives; FP, false positives; TN, true negatives.

**Fig 3:**
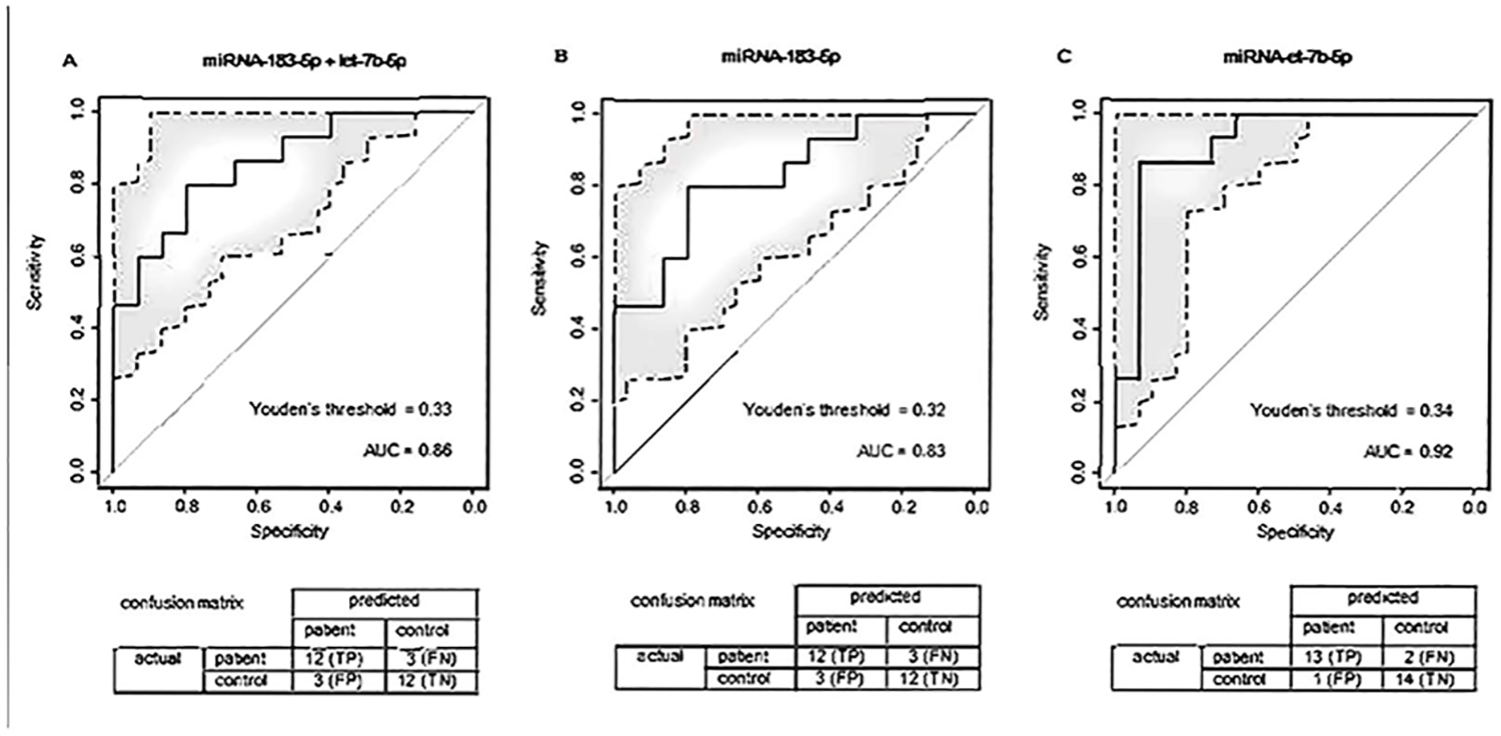
ROC curves of patients with untreated UIA and controls. ROC, Receiver operating characteristic; aSAH, aneurysmal subarachnoid hemorrhage; UIA, unruptured intracranial aneurysm; AUC, area under the curve; TP, true positives; FN, false negatives; FP, false positives; TN, true negatives.

Fig 2 shows the ROC curves of patients with aSAH and controls while Fig 3 shows these curves of patients with untreated UIA and controls. These figs include the optimal Youden’s J thresholds and confusion matrices. In distinguishing aSAH patients from controls, AUC was 0.80 (95% CI 0.63–0.97) for miRNA-183-5p, and 0.74 (95% CI 0.55–0.94) for miRNA-200a-3p. Combining both these two miRNAs in a single model did not improve the performance for risk associations (AUC of 0.79 (95% CI 0.61–0.96); Fig 2). In distinguishing patients with untreated UIA from controls, the AUC was 0.83 (95% CI 0.55–0.94) for miRNA-183-5p and 0.92 (95% CI 0.81–1) for miRNA-let-7b. Again, combining these two miRNAs in a single model did not improve the performance for risk associations with an AUC of 0.87 (95% CI 0.73–0.99; Fig 3**)**.

### Target genes

MiRNAs-183-5p, miRNA-200a-3p and miRNA-let-7b-5p have 1579 unique target genes and 15 out of 109 candidate genes (13.8%) previously described to be involved in IA development and rupture in GWAS and GWES are among these target genes (Table 4). The probability of these 15 genes overlapping purely by chance (p-value of the observed outcome) is 0.02, which would constitute a statistically significant finding, taking into account the conventional level of significance of 5%.

**Table 4:** Comparison of the target genes of the miRNAs with a difference in levels and candidate genes for IA as identified in genes found in GWAS [15,16] and GWES [17].

		miRNA 183-5p	miRNA 200a-3p	miRNA let-7b
GWAS locus[17–18]	10q24.32	*PCGF6*		*TAF5*
	13q13.1		*PDS5B*	*STARD13*
	18q11.2			*RIOK3*
	4q31.23	*TMEM184C*		
GWES[19]		*RUNX1T1*	*CXCL12*	*IGF1* *IL6*
				*COL1A1*
				*COL1A2*
				*COL3A1*
				*COL5A1*
				*COL5A2*

IA, intracranial aneurysms; GWAS, genome wide association study; GWES, genome wide expression study; *PCGF6*,polycomb group ring finger 6; *TAF5*, TATA box binding protein (TBP)-associated factor 5; *PDS5B*, Sister chromatid cohesion protein PDS5 homolog B, *STARD13*, StAR-related lipid transfer domain protein 13; *RIOK3*, RIO kinase 3; *TMEM184C*, transmembrane protein 184C; *RUNX1T1*, RUNX transcription factor 1; *CXCL12*, C-X-C motif chemokine 12; *IGF1*, insulin-like growth factor 1; *IL6*, Interleukin 6; *COL1A1/1A2/3A1/5A1/5A2*, Collagen, type 1/3/5, alpha 1/2)

## Discussion

We found that miRNA-183-5p, miRNA-200a-3p and miRNA-let-7b-5p have a difference in levels in blood of IA patients as compared to controls. These miRNAs had good to excellent discriminating power for distinguishing IA patients from controls.

In the replication study, miRNA-183-5p level appeared to be significantly decreased in both aSAH patients and patients with untreated, UIA. MiRNA-let-7b-5p was only evaluated in patients with untreated UIA patients and found to be decreased in these patients. MiRNA-200a-3p level was increased only in aSAH patients, and not in IA patients, which might suggest that miRNA-200a-3p influences the risk of aneurysmal rupture.

Of the 109 genes previously described to be involved in IA development and rupture in GWAS and GWES [17–19] 15 genes appear to be targeted by miRNA-183-5p, miRNA-200a-3p or miRNA-let7b-5p, identified in our study. This proportion of targeted genes was found higher than would be expected by chance. These miRNAs target genes that belong to important biological pathways implicated in IA pathogenesis, including cell proliferation (*IGF1*, *PCGF6*, *RUNX1T1*, *TMEM184C*, *STARD13* and *PDS5B*), extra cellular matrix composition (*COL1A1*, *COL1A2*, *COL3A1*, *COL5A1* and *COL5A2*) and inflammation (*CXCL12*, *IL6*).[22]

Interestingly, miRNA-let-7b was previously found to be decreased in ruptured IA samples as compared to control samples (being superficial temporal arteries) [23] although two other studies on miRNA levels in IA samples did not confirm these results.[24–25] MiRNA-let-7b was also found decreased in plasma of patients with another type of aneurysm (abdominal aortic aneurysms) [26] although again these results were not confirmed in two comparable other studies.[27–28] Besides these three studies on miRNAs levels in IA tissue,[24–25] we identified two previous studies also analyzing miRNAs in blood of patients with IA.[29,30] In these studies miRNA-183-5p, miRNA-200a-3p and miRNA-let- let7b-5p were not found to have a difference in levels.[29,30] In the first PCR array study, 86 miRNAs with a difference in levels were identified when analyzing three different subgroups of six patients each (two groups with UIA patients with and without irregular shape and a third group with ruptured IA patients) and comparing them to the controls. The 86 miRNAs were not replicated in an independent cohort which may be explained by the relatively small sample size of the subgroups analyzed in the initial discovery phase.[29] The second microarray study identified 20 miRNAs with a difference in levels in both 20 aSAH and 20 UIA patients as compared to 20 controls.[29] Subsequently, a second independent microarray study was performed in 93 IA patients (with unknown rupture status of the IA) and 50 controls which identified 99 miRNAs with a difference in levels. Of these, 99 miRNAs, 12 (12%) were also found in the initial study.[30] These 12 miRNAs do not include the three miRNAs with a difference in levels (miRNA-183-5p, miRNA-200a-3p and miRNA-let-7b) identified in our study. In the two independent microarray studies, however, no correction for multiple testing was applied and no replication experiments were performed which may explain the differences in results.[30] In animal models of IA miRNA-183-5p, miRNA-200a-3p and miRNA-let-7b-5p were also not found to have different levels as compared to controls animals.[31–32]

A strength of this study is that the miRNAs identified in the discovery cohort were tested in a separate cohort for replication by RT-qPCR. Furthermore, blood samples were obtained at the same time and under the same circumstances for all participants. Our study also has limitations that need to be considered. The first limitation concerns the patient selection in the discovery group. This group consisted of aSAH patients of whom only a subgroup had additional UIA, and not of patients with UIA alone; this may have left additional miRNAs, associated with UIA, undetected in our study. However, the use of patients with prior aSAH can be justified, as IA appears to be a continuous disease process in the intracranial vessel wall: patients with prior aSAH are still at risk of developing new aneurysms, and of growth of already present unruptured IA.[33] Another limitation of our study is the limited sample size used in both the discovery and replication phases. Further replication studies should confirm our results since is important to realize that we did not correct for multiple testing although the miRNAs identified in the discovery cohort were tested in a separate cohort for replication. However, of the five miRNAs selected for replication two of them did not replicate which confers to a false positive rate of 40% which may be considered high. However this rate is better than the false positive rates of around 87% as found in the two previous studies analyzing miRNAs in blood of patients with IA.[29,30]

Future studies are also needed to investigate whether the miRNAs identified in our study can be applied in a diagnostic test to identify individuals at high risk of IA development and its subsequent rupture. These individuals include those first-degree relatives of familial aSAH patients at high risk of aneurysm development who will benefit most from early detection and preventive treatment of IA. A first step towards the development of such a new diagnostic test for these first-degree relatives may be a retrospective study on a cohort of first-degree relatives who have been screened for IA in the past. In this cohort, the levels of the identified miRNAs can be compared between first-degree relatives, in whom an IA was identified at screening, to those in whom no IA was found, to see if these miRNAs are able to distinguish between the two groups. Furthermore, the therapeutic role of the miRNAs associated with aneurysm development and/or rupture should be explored.

In conclusion, we identified the three specific circulating miRNAs miRNA-183-5p, miRNA-200a-3p and miRNA-let-7b that are able to discriminate between IA patients and controls. Future studies are needed to investigate whether these miRNAs may be used as biomarkers for identifying individuals at high risk of IA development and its subsequent rupture.

## Supporting information

S1 File Database containing microRNA levels of discovery phase using PCR array and of replication phase using real-time (RT) PCR. (XLSX)
